# Quality of life and regional economic development: Evidence from China

**DOI:** 10.1371/journal.pone.0298389

**Published:** 2024-05-16

**Authors:** Yuhu Cui, Hu Tian, Dong An, Yonghua Jia

**Affiliations:** 1 School of Business, Shandong University, Weihai, China; 2 College of Management, Ocean University of China, Qingdao, China; 3 School of Bohai, Hebei Agricultural University, Baoding, China; 4 Research Center for Marine Economy and Coastal Economic Belt, Hebei Normal University of Science & Technology, Qinhuangdao, Hebei, China; Shenzhen University, CHINA

## Abstract

With the development of China’s economy entering a new stage, the quality of life, which centers on the well-being of residents, provides an essential hand in promoting the transformation of the regional economy from high-speed development to high-quality development. Based on a panel threshold regression model, we examine in this paper whether quality of life helps regional economies realize developmental convergence. The research shows that: (1) The quality of life overall can promote regional economic development and passes the series test with relatively robust results. (2) The quality of life has a non-linear effect on regional economic growth, which is mainly manifested in the fact that the impact is more significant in regions with higher levels of quality of life and weaker in regions with lagging quality of life and may widen the gap between regions at the same time. (3) We categorize the study regions to test further regional heterogeneity based on regional location and development status. At the *Quality of Life Level-I* regions, their influence on economic development has a more substantial pulling effect. Therefore, each region should seize the strategic opportunity to improve the quality of life, focus on the balanced development of the quality of life, strengthen policy support and social security, and strive to promote the coordinated development of China’s regional economy.

## 1. Introduction

Improving the quality of life is one of the significant goals of Chinese-style modernization. As China’s economic development enters a new normal, people’s concern for the quality of life has gradually increased because of the environmental pollution and various social problems brought about by the previous rapid economic development (Haq and Zia, 2013) [1]. Thus, enhancing the quality of life is closely related to people’s lives and the fundamental starting point and landing point of regional economic development. China’s focus on the quality of life, the formulation and release of the “*China Quality Ten-Year Agenda*,” will continue to improve the living standards of nationals, fully reflecting the trend of “people-oriented, people-service, green development.” In this context, the research on the impact of quality of life on regional economic development is of significant theoretical significance and practical value in grasping the opportunity period of modernization and promoting coordinated regional development.

In terms of traditional economic development theories and influencing factors, they have not been effective in capturing the unexplained parts of economic growth (Fedderke and Klitgaard, 1998) [2]. Therefore, some scholars advocate finding additional or alternative factors to explain the intangible factors. For example, quality of life is one of the most critical factors influencing economic decisions, and high quality of life, favorable environmental location, and cultural atmosphere, as well as good infrastructure, are the critical factors promoting regional economic development and creating employment opportunities (Testa and Simonson, 1996) [3]. Throughout the history of the developed world, quality of life has always been given a prominent place. In Asia, Singapore has been recognized as the “*Best City in Asia*” in terms of quality of life, according to Mercer, a global human resources consulting firm, and as the “*Happiest Country in Southeast Asia*” in the *World Happiness Report*. As a result of this high level of quality of life, Singapore has become a popular destination for international immigrants, bringing with it capital, technology, management knowledge and access to international export markets, providing a substantial “invisible support” for Singapore’s economic development (Mahirah et al., 2020) [4]. In Europe, the United Kingdom also attaches great importance to developing quality of life. The country’s leading human resources organization, Totaljobs, surveyed and ranked the quality of life in 15 major cities in the UK. The survey included indicators such as cost of living, house price to income ratio, rental costs, safety, healthcare quality, air and water quality, and green space quality. The results indicate that Edinburgh has the highest quality of life score, with 97% of Edinburgh residents being delighted with their lives and scoring the highest regarding healthcare services and air quality (Mõttus et al., 2012) [5]. This shows that along with the social and economic development, people’s demand for the living environment and social security has been increasing to realize their value through relocation, which brings more development resources to the relocation area and, to a certain extent, boosts the regional economic development. However, as the world’s largest developing country, China faces many difficulties and challenges in economic transformation and development. So, is China’s quality of life a “booster” or a “roadblock” to regional economic development? What is its impact on regional economic development? Is there any heterogeneity depending on factors such as regional location? By clarifying the above questions, we hope to provide new perspectives and new ideas for the high-quality development of China’s regional economy.

The research on the relationship between quality of life and economic development is a topical concern of scholars at home and abroad. The existing literature mainly focuses on the following aspects: On the one hand, in terms of the impact of economic development on the quality of life, modern economic growth certainly improves the quality of life, and the quantities of food, clothes, and shelter needed by the people obviously increase, and the standard of living develops qualitatively (Max-Neef, 1995; Easterlin and Angelescu, 2011) [6, 7]. Esposto et al. (1999) [8] analyze the impact of economic freedom on the quality of life and find that higher economic freedom will significantly improve the quality of life in each country. Nevertheless, regional economic development will eventually reach a “critical point”, that is when economic development crosses the “critical inflection point value”, it will put pressure on the local social development and resources and environment, causing an increase in the cost of living and an overall decline in the quality of life, which will to a certain extent affect the regional economic development. To a certain extent, this will negatively impact regional economic development (Felce and Perry, 1995) [9], such as the United States “Silicon Valley” in the economic development of the “leap,” which led to its regional traffic jams, environmental degradation, housing prices, the quality of life and other aspects of the conditions of the downward trend, which is also to certain extent constraints on economic development. Therefore, the quality of life is subjective and social; it reflects the characteristics of the outcomes of a particular industry and will inevitably impact economic development (Evandrou and Glaser, 2004) [10].

On the other hand, regarding the impact of quality of life on economic development, quality of life is becoming an essential concept in the theoretical and empirical field of economics. Especially in city economics, quality of life factors can significantly contribute to the location decisions of households and firms. Based on the Carlino-Mills regional growth model, existing studies have integrated quality of life and comfort into the model, and the results show that there is a predictable relationship between convenience of life and local economic performance (Deller et al., 2001) [11]. Some scholars have demonstrated that national living comfort is positively correlated with economic development to a certain extent in developing countries (Lambiri et al., 2007) [12]. However, the time series data do not support this view. In the case of China, the Quality of Life in Chinese Cities group has taken the lead in measuring the quality of life in major cities, which has led to the conclusion that, despite the improvement in the quality of life of the residents in the process of rapid economic development, there is still a particular gap between their actual feeling of life and their subjective well-being (Liu, 2006) [13]. Regarding the construction of quality of life indicators and regional spatial distribution, the quality of life theory is used as a guide to establish a mechanism for the two-way influence of rural space and quality of life, which verifies the relationship between rural spatial development and farmers’ living standards in various aspects. It effectively promotes the development of the countryside (Vaishar et al., 2018) [14]. Meanwhile, during the research process, Li and Wang (2013) [15] initially constructed an evaluation index system for city quality of life, took Chinese region as the main body of the research, gave an economic elaboration on the differences in regional spatial patterns, and emphasized the need to adhere to the concept of people-oriented, promoting the synergistic development of regional economy and quality of life.

By combing through the relevant literature, we found that the current research still has the following shortcomings: First, most of the existing research focuses on the effect of economic development on quality of life, but the research on the impact of quality of life on the regional economy in the new development stage is relatively weak, and most of them are carried out at the national or provincial level. Second, the standard measurement of quality of life still needs to be unified. As the economy and society continue to develop, the relevant indicators must be further updated. Third, the literature mainly adopts the spatial distribution form to show the distribution characteristics of quality of life and less involves the study of city heterogeneity of quality of life on regional economic development. Different regions have their features derived from the differences in their location advantages, economic development levels and policy backgrounds.

Accordingly, this paper will use the panel data model and threshold regression model to analyze the above issues empirically. To begin with, according to *the Report on the Quality of Life in Chinese Cities*, the quality of life index of 35 major cities in China from 2012 to 2019 is selected as the data support for the core explanatory variables. In addition, we apply the panel regression method to analyze the relationship between the quality of life and regional economic growth to verify the role of the quality of life in influencing regional economic development. Moreover, the panel threshold regression model is used to examine further the non-linear impact of quality of life on regional economic development and to identify the extent of the quality of life on economic development in different regions to derive its convergence. Finally, for the research conclusions, we provide relevant policy recommendations from the perspective of coordinated development of quality of life and regional economy. This paper may bring the following marginal contributions: (1) to explore the relationship between regional quality of life and economic development in China at the city level, using the existing official quality of life indexes as the data support; (2) to further clarify the mechanism of the impact of quality of life in different regions on regional economic development, providing a new way of thought on high-quality development of China’s economy; (3) to use the threshold regression model as an essential tool to explore the non-linear impact of quality of life on regional economic development and to provide a theoretical basis and practical reference for the synergistic development of the inter-regional economy.

## 2. Theoretical basis and research hypothesis

**Fig 1:**
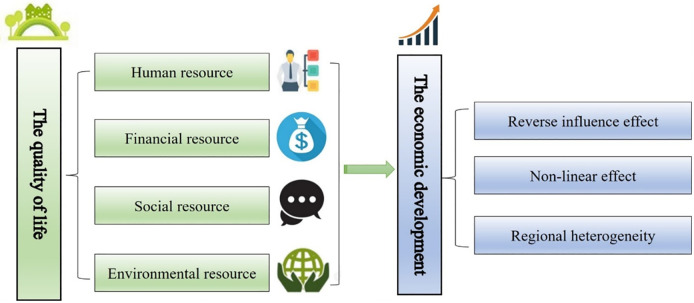
Mechanism of quality of life impacts on the regional economy.

In theoretical terms, quality of life usually refers to an outcome of the development of social policies and programs and is a comprehensive concept (Kaplan and Ries, 2007) [16]. While developing the economy, modern countries also need to emphasize social equity, improve education, protect the ecological environment, and formulate policies based on people’s needs and expectations to improve the overall quality of life and reverse economic growth. The concept of quality of life appeared first in the book *The Affluent Society* by American economist John Kenneth Galbraith (1998) [17]. Since then, quality of life has gradually become a specialized research field, and most scholars have studied its measurement methods and indicator systems. Foreign research on quality of life has been conducted earlier, mainly on medicine and sociology. It is considered that quality of life is an individual’s total social welfare (Lamers et al., 2005) [18], perception of one’s situation and the need for social development, which in turn affects the development of the economy. In China, there are three definitions of quality of life: first, from a subjective point of view, quality of life is the satisfaction of people’s subjective perception of life. Second, from the objective point of view, the quality of life reflects the satisfaction of people’s living conditions in objective aspects, such as clothing, food, housing, transportation, and use. Third, since subjective and objective perspectives, we consider life as the degree of adequacy of society to improve people’s perception of life and the degree of fulfillment of the national life needs (Shek et al., 2005) [19]. Moreover, this paper regards the quality of life as a whole system, mainly from economic development, urban construction, social security and so on (see Fig 1).

At the level of living needs, a high level of quality of life means a broader demand for various goods and services, which will further stimulate the growth of the market and provide a new impetus for the regional economy. For the labor market, a stable and positive quality of life will help attract talent in various industries, thus further promoting scientific and technological innovation, upgrading the industrial structure, and laying a solid foundation for the regional economy (Terziev, 2009) [20]. Regarding social stability, by strengthening the policy effects of education, healthcare and environment, each region will gradually eliminate people’s potential risk concerns, improve personal security, promote industrial change, and offer a stable environment for regional economic development. Based on this, this paper proposes hypothesis H_1_:

**H**_**1**_**:** Quality of life is a new driving force for economic development, and it effectively promotes the level of regional economics.

The impact of quality of life is strongly dependent on a favorable environment and ecology. Capital is a critical element in raising the level of city infrastructure, completing the social security system, and eliminating the “quality of life gap.” The development of the city’s quality of life can create a favorable business environment, attract more enterprise capital and high-end talents and other resources to gather, and then provide a vital prerequisite for economic development. There is a consensus that enterprise development and talent attraction are essential factors in improving economic quality (Shapiro, 2006) [21]. Quality of life may have a non-linear impact on economic development. If the quality of life exceeds a certain threshold, it will have a positive impact on economic development. When the quality of life is lower, the potential pull effect is limited by the need for a comprehensive infrastructure and social security system and the lengthy investment cycle of related activities, resulting in relatively low industrial returns. With the development of Chinese society and economy, under the demand and drive of the interests of multiple subjects, the quality of life continues to improve. Resources such as capital, technology and talents continue to focus on the reverse role of the economy, which will be more obvious. The scale effect of the quality of life is gradually highlighted. So, we put forward hypothesis H_2_:

**H**_**2**_**:** The quality of life empowers regional economic development with a non-linear impact as the infrastructure improves and the level of social security rises.

The quality of life varies significantly between regions in China, owing to geographic location, economic and social level, and other factors. Quality of life empowerment effect requires certain preconditions to be supported (Parushina et al., 2019) [22]. On the one hand, different regions of the city’s economic development of the original situation and infrastructure construction and other aspects of the existence of a particular gap, making the living environment and other preconditions to support the different strengths, resulting in the quality of life brought about by the economic impact of the effect of the existence of heterogeneity; on the other hand, the existing research pointed out that the city administrative level, the coastal location of the province, such as the influence of the factors, the quality of life and the economic development of the existence of a more significant impact. For example, the higher the administrative level of coastal provinces and cities (coastal capital cities), the priority of various resources, financial and policy support, and the higher the degree of impact of quality of life on economic development. Based on this, this paper raises a hypothesis H_3_:

**H**_**3**_**:** The effect of quality of life on economic development is regionally heterogeneous, and the empowering impact is more significant in cities with higher quality of life.

## 3. Methodology

### 3.1 Model setting

To investigate further the impact of quality of life on regional economic growth and whether there are differences in economic growth in different regions under the effect of quality of life. This paper will use a panel data model and a threshold model to conduct an empirical study. The following panel data models are shown:

$$Qgdp_{it}=\alpha_{0}+\alpha_{1}\text{ln}\;life_{it}+\beta X_{it}+\mu_{i}+\delta_{t}+\varepsilon_{it}$$ (1)

where *Qgdp*_*it*_ represents the GDP per capita of region *i* in period *t*, and ln *life*_*it*_ shows the quality of life level of region *i* in period *t*. *X*_*it*_ is a series of control variables indicating other factors affecting the regional economic development level, which mainly include total population, education cost expenditure, foreign trade dependence, urban CPI, urban greening rate and water resources situation, etc.; *μ*_*i*_ is an individual fixed effect, *δ*_*t*_ is the time fixed effect, and *ε*_*it*_ is the residual term.

The effect of quality of life on regional economic development can be estimated by Eq (1) overall. However, since there may be nonlinear effects of quality of life on regional development, this paper further develops a panel threshold model of the nonlinear relationship between quality of life and regional economic development as follows:

$$Qgdp_{it}=\alpha_{0}+\alpha_{1}\text{ln}\;life_{it}I(p_{it}\leq \gamma )+\alpha_{2}\text{ln}\;life_{it}I(p_{it}>\gamma )+\beta X_{it}+\mu_{i}+\varepsilon_{it}$$ (2)

Where *Qgdp*_*it*_, ln *life*_*it*_ and *X*_*it*_ are consistent with the meaning in Eq (1). *p*_*it*_ is the threshold variable, and the threshold variable can be taken as the level of quality of life or the degree of regional development in the empirical estimation below. *γ* is the threshold value, *α*_1_ and *α*_2_ indicate the degree of impact of quality of life on regional economic development under different threshold values. If they are equal, it means that there is no non-linear effect of quality of life on regional economic development; if *α*_1_ > *α*_2_, it indicates that quality of life has a more significant contribution to its economic development in regions where the quality of life or regional economic development is lagging behind, and vice versa. *μ*_*i*_ is the individual fixed effect, and *ε*_*it*_ is the residual term.

### 3.2 Variable selection

#### The explained variable

In this paper, regional GDP per capita is used to indicate the level of regional economic development, and the data are mainly sourced from *the China City Statistical Yearbook* of the past years. GDP per capita is one of the most important indicators for assessing the level of regional economic development, which refers to the indicator obtained by dividing the total regional GDP by the total population at a specific time (Pastor et al., 2018) [23]. It tends to be correlated with several variables, such as population size and fiscal policy.


$$GDP_{per}=GDP_{total}/POP_{ave}$$ (3)

#### Core explanatory variable

Based on the average value of subjective and objective quality of life indices in the “*Report on the Quality of Life in Chinese Cities*” compiled by the China Institute of Economic Experimentation as an explanatory variable. The subjective quality of life index takes the survey of residents’ happiness as the basis, involving residents’ income, cost of living, medical protection, and convenience of life, etc.; the objective quality of life index is shown by the index of socio-economic data, including per capita wealth, inflation, Engel’s coefficient, and other aspects. This index generally reflects all aspects of the quality of life of urban residents in China at this stage. We used the mean of the subjective and objective indices as the core explanatory variable.


$$life_{index}=(Sub_{index}+Obj_{index})/2$$ (4)

#### Control variables

Education expenditure (*edu*), financial education expenditure which not only promotes economic growth, but also has certain macro effects on employment and inflation. We use the expenditure on education in each region and logarithmic treatment (Hao et al., 2022) [24]. Foreign trade dependence (*fore*), this indicator reflects the level of a country’s economic development and the degree of contribution to the participation in the world economy. It is calculated as the ratio of total imports and exports to GDP, with total imports and exports converted to RMB based on the prevailing interest rate (He and Zhang, 2010) [25]. Consumer Price Index (*CPI*), which is based on the consumer price index to better observe its impact on the quality of life of the population and the economic development of the country (Wang et al., 2016) [26]. Population (*pop*), which plays an essential role in economic development within the scope of science, is expressed as the logarithm of the population of each region (Du et al., 2022) [27]. City greening rate (*green*), which is a key indicator of the quality of life of residents (Feng et al., 2021) [28], is conducive to improving the quality of the environment, promoting the optimization of the industrial structure, and enhancing the vitality of economic development. Total social retail sales (*str*), the concentration of the level of consumption of residents, from the side of the impact of consumption on the economy, and take the logarithm (Nazarenko and Zhong, 2020) [29]. Sewage treatment rate (*tsrs*), regional sewage treatment is an important reflection of the level of social services and has a certain potential impact on economic development (Yang et al., 2020) [30].

The above data are obtained from *the China Statistical Yearbook* and *the China Urban Statistical Yearbook*. Table 1 shows the results of descriptive statistics of each variable. For individual regions for individual years missing data values, the use of interpolation method to supplement.

**Table 1:** Descriptive statistics of variables.

Variable	Obs	Mean	Std. Dev.	Min	Max
*Qgdp*	280	9.112	3.304	3.513	20.348
*lnlife*	280	4.049	0.102	3.879	4.323
*lnedu*	280	14.149	0.829	12.139	16.245
*fore*	280	0.307	0.281	0.019	1.405
*lncpi*	280	4.626	0.008	4.603	4.658
*lnpop*	280	6.445	0.687	5.085	8.136
*lngre*	280	3.712	0.097	3.302	4.120
*lntsrs*	280	4.510	0.086	4.119	4.675
*lnstr*	280	17.107	0.817	14.966	18.881

## 4. Empirical results

### 4.1 Analysis of benchmark results

To mitigate the problem of heteroskedasticity, we take natural logarithms for all variables in the regression. Table 2 presents the estimation results of the overall quality of life impact on regional economic development. Model 1 uses the least squares (OLS) method for regression estimation; Models 2 and 4 are the results of assessment using the fixed-effects model, which considers the city fixed effects and the city and time two-way fixed effects, and Models 3 and 5 use the random-effects model, which also considers the city fixed effects and the city and time fixed effects, respectively. The decidable coefficients R^2^ of the above five models are all greater than 0.5, which proves that the overall fitting degree of each model is better, and the estimation results can be further analyzed.

**Table 2:** Baseline regression results.

Variable	Model 1	Model 2	Model 3	Model 4	Model 5
	OLS	FE	RE	FE	RE
*lnlife*	4.958*** (1.385)	0.501* (0.219)	2.095* (1.052)	1.213 (3.421)	4.804* (3.227)
*lnedu*	4.655*** (0.351)	5.572*** (0.551)	5.052*** (0.453)	4.470*** (0.635)	4.244*** (0.464)
*fore*	1.081** (0.522)	-0.065 (0.650)	0.584 (0.586)	0.068 (0.559)	0.722* (0.509)
*lncpi*	5.668 (13.694)	3.929* (2.923)	5.191 (9.209)	-2.905 (8.041)	0.012 (8.194)
*lnpop*	-3.923*** (0.368)	-0.031 (1.042)	-3.693*** (0.556)	-0.450 (0.891)	-3.008*** (0.540)
*lngre*	-0.694 (1.276)	0.138 (1.234)	0.247 (1.214)	0.301 (1.061)	0.260 (1.046)
*lntsrs*	-0.919 (1.401)	-0.994* (0.658)	-0.712 (1.316)	-0.537 (1.171)	-0.571 (1.154)
*Cons*.	-71.375 (65.089)	-85.757* (42.643)	-68.946* (43.901)	-42.431* (32.431)	-50.579* (41.192)
City FE	N	Y	Y	Y	Y
Year FE	N	N	N	Y	Y
Obs.	280	280	280	280	280
R^2^	0.647	0.541	0.628	0.682	0.667

Note: The robust standard error is in brackets.

***p < 0.01,

**p < 0.05,

*p < 0.1, the same as below.

In Model 1, the coefficient of the quality of life variable is 4.958. It is significant at the 1% level, indicating that for every 1% increase in the quality of life level, the absolute value of GDP per capita at this time will increase by 0.049 units. For model 2-model 5, the corresponding quality of life coefficients are all positive, indicating that whether using the fixed effects model or random effects model, as well as whether the city and time effects are fixed in both directions, the promotion of the quality of life for the regional economic development is more robust, which to some extent verifies the hypothesis H_1_. The quality of life is a significant manifestation of residents’ survival and development and has a complex impact on economic development. Those regions that emphasize quality of life tend to provide a superior natural environment, favorable education, healthcare, and other public services, which can enhance residents’ happiness, promote technological progress and consumption upgrades, and contribute to the sustainable development of the regional economy (Ndaguba et al., 2018) [31]. Chengdu, China, has been awarded “China’s Happiest City” for 13 consecutive years. It attaches great significance to the quality of life of its residents. It has continued to make efforts in the areas of innovation, green and low-carbon, and people’s well-being, as well as carrying out activities to build a high-quality quality of life in the city and attracting more enterprises and human resources, etc., resulting in an economic output that has exceeded RMB 2 trillion, and a total amount of foreign trade and import and export that exceeds RMB 830 billion, which adds more impetus to the development of the region’s economy.

From other control variables, the investment in education is significantly positive at the 1% level in all five models, indicating that the investment in education can contribute substantially to the improvement of per capita GDP. The impact of education on economic development is multifaceted, as it improves the quality and skill level of workers, promotes scientific and technological innovation and technological progress, enhances employability and entrepreneurship, promotes economic development, and improves the quality of life of the people and the level of social development (Kireenko and Nevzorova, 2015) [32]. The coefficients of foreign trade dependence and consumer price index are mostly positive in the five models, but the coefficients in some models are weakly significant. The reason is that China’s economy has gradually entered a new normal, the domestic market consumption potential is constantly "power," and at the same time by the downward pressure of the economy and other external shocks, the role of domestic consumption to drive economic growth is gradually highlighted, so the pulling force of foreign trade growth on economic development is gradually declining, which will lead to a new round of regional changes.

From the population situation, the coefficient result is negatively correlated, but the result is not significant in Model 2 and Model 4. The reason for concern is that from the economic perspective, population growth will squeeze out more living resources, thus leading to a decline in the level of people’s living environment and bringing about specific negative impacts on economic development. Thus, the effect of promoting economic growth through population increase is gradually weakening, which is a deviation from the expected results. In terms of city greening rate and domestic sewage treatment, the estimation results and significance of both are not very significant. However, from the perspective of real life, improving city greening rate and sewage treatment is an important performance to ensure the happiness of residents’ lives. However, as a control variable, its direct impact on economic development is weak, which leads to the regression estimation results are not very robust (Frölich, 2008) [33].

### 4.2 Endogenous problem

This paper has a potential endogeneity problem based on its modeling. On the one hand, there may be reverse causality between the quality of life and regional economic development; on the other hand, there may be measurement bias in the quality of life indicator system used in this paper, leading to correlation between regional economic development and unobservable factors affecting quality of life. Therefore, by following Bellemare et al., (2017) [34], we select the lagged period product of the quality of life variable and its first-order difference as the instrumental variable and estimate the model by adopting the two-stage least squares (2SLS) method and generalized matrix estimation method (GMM).

We take the above treatment for the following reasons: First, the city quality of life index comes from the synthetic mean of 35 cities, and its trend will not be significantly affected by individual cities, and the difference terms can be considered exogenous concerning respective cities. Second, the quality of life level of individual cities may be affected by other unobserved shocks. However, if we control for the impact that such shocks bring to the overall index, we consider the instrumental variables to be valid. Furthermore, since the number of variables is reduced from 280 to 245 due to the differential treatment of the variables, Table 3, columns 2–4 reports the second-stage estimation results of the instrumental variable method under different estimation methods and from the effects of its correlation test, the model passes the test of under-identification of instrumental variables, the examination of weak instrumental variables, and the difficulty of endogeneity, which indicates that the instrumental variables in this paper are more reasonably selected. From the estimation results, the quality of life coefficients are all significantly positive, showing that the quality of life will empower economic development and will promote the regional economic growth, which is consistent with the benchmark results.

**Table 3:** Estimated results after adding instrumental variables.

Variable	2SLS		GMM
	FE	RE	
*lnlife*	0.475* (0.225)	0.501*** (0.142)	0.473* (0.245)
*Cons*.	-35.181** (20.251)	-13.215*** (5.321)	-10.250*** (5.535)
*Contral*	Y	Y	Y
City FE	Y	Y	Y
Year FE	Y	Y	Y
Kleibergen-Paap rk LM	25.218 (0.000)	21.154 (0.000)	13.254 (0.005)
Kleibergen-Paap rk Wald F	35.843 (18.128)	30.982 (14.852)	53.896 (16.359)
Obs.	245	245	245

### 4.3 Non-linear model estimation

Considering the theoretical analysis above, this paper will use the threshold regression model to estimate Eq (2) by setting a single threshold, double threshold, and triple threshold to verify further the non-linear impact of quality of life on regional economic development. To avoid the subjective bias caused by artificially dividing the sample interval, we adopt the self-sampling method (Bootstrap) to sample 300 times to get the test results repeatedly (Lu and Li, 2020) [35]. As shown in Table 4, by comparing the F-value with the critical value, we know that the threshold variables of regional economic development and quality of life pass the single-threshold significance test at the 1% confidence level, and the corresponding threshold values are 12.071 and 4.239.

**Table 4:** Threshold effect test.

Threshold variable	Num. threshold	Threshold value	F-value	P- value	10% critical value	5% critical value	1% critical value
*Qgdp*	Single threshold	12.071	85.012***	0.000	15.325	20.211	27.652
	Double threshold	-	43.251	0.156	18.526	25.915	34.189
	Triple threshold	-	8.35	0.236	37.713	47.652	60.159
*lnlife*	Single threshold	4.239	64.224***	0.000	16.523	21.532	30.178
	Double threshold	-	14.105	0.086	14.105	17.258	23.354
	Triple threshold	-	11.342	0.154	34.726	48.942	61.852

Therefore, according to the results of the threshold effect test, we estimate the regression of the model again from different situations (see Table 5). In Model 6, the threshold variable is regional economic development (*Qgdp*), corresponding to the threshold value 12.071. Currently, when the regional economic development situation is lower than 12.071, the coefficient corresponding to the quality of life is 0.065. When the *Qgdp* is more significant than 12.071, its corresponding coefficient is 0.090, and both coefficients have a certain degree of significance. By comparing the coefficients of the two situations, the latter is larger than the former, which indicates that in regions with a better level of economic development, the pulling effect of quality of life on regional economic development is more prominent. From another point of view, relative to the lower level of economic development in the region, the quality of life will also stimulate economic development and promote regional consumption and industrial upgrading. However, its growth rate is still lower than that of the better level of economic development in the region, indicating that the quality of life is not conducive to the economic development of the backward region and even leads to the development gap between the two widenings, which will bring more fundamental problems. This also confirms the hypothesis H_2_, which is that the quality of life will have a non-linear impact on regional economic development and may exacerbate the development imbalance between regions. According to *the Better Life Index for China’s Major Cities Report*, eight of the top ten cities are in developed eastern regions. Eastern cities have a high overall index of living well in seven areas: employment and wages, residents’ livelihood, social security and social services, public facilities, ecological environment, health, and tourism. Specifically, Eastern China has a well-developed infrastructure and economic system, abundant human resources, scientific and technological innovation capacity, and advanced international trade, which makes its quality of life a more vital driving force for economic development.

**Table 5:** Panel threshold estimation results.

Variable	Model 6 (*Qgdp*)		Variable	Model 7 (*lnlife*)	
	*Qgdp*<12.071	*Qgdp*≥12.071		*lnlife*<4.239	*lnlife*≥4.239
*lnlife*	0.065* (0.034)	0.090* (0.065)	*lnlife*	0.076* (0.045)	0.088* (0.071)
*lnedu*	1.720*** (0.552)	1.720*** (0.552)	*lnedu*	2.718*** (0.677)	2.718*** (0.677)
*fore*	0.110 (0.488)	0.110 (0.488)	*fore*	-0.039* (0.611)	-0.039* (0.611)
*lnpop*	0.026 (0.776)	0.026 (0.776)	*lnpop*	0.126 (0.964)	0.126 (0.964)
*lngre*	0.121* (0.928)	0.121* (0.928)	*lngre*	0.280 (1.164)	0.280 (1.164)
*lntsrs*	3.315*** (0.489)	3.315*** (0.489)	*lntsrs*	3.257*** (0.604)	3.257*** (0.604)
*Cons*.	-91.373** (45.104)	-91.373** (45.104)	*Constant*	-93.150*** (12.329)	-93.150*** (12.329)
R^2^	0.736		R^2^	0.604	
Obs.	280		Obs	280	
Threshold P-value	0.000		Threshold P-value	0.105	

When the threshold variable is quality of life, the corresponding threshold value is 4.239. At this point, the coefficients of the two are 0.076 and 0.088. The latter is larger than the former, which shows that regions with a higher quality of life have a more significant role in regional economic development. In contrast, regions with a poorer quality of life have a lesser role in pulling economic development, indicating to some extent that the level of the quality of life has a particular disadvantage for latecomers. At the same time, its threshold P-value is more significant than 10%, which is statistically weak. High quality of life has a potential advantage in attracting talent and enterprise investment, and the economic development of regions with comparative advantages is self-reinforcing through the advantage of precedence and scale efficiency (Esmaeilpoorarabi et al., 2016) [36]. According to China’s livability study, Hangzhou is ranked No. 1, with a pleasant natural environment, good infrastructure, and a favorable business environment (Internet economy, etc.), which provide essential prerequisites for strong economic development.

### 4.4 Robustness tests

To ensure the reliability of the research conclusions, this paper adopts the following ways to test the robustness: Replace the explained variable and Convergence effect test.

#### 4.4.1 Replace the explained variable

According to the existing research, we change the regional economic development indicator into the GDP growth rate; the growth rate of GDP can reflect the economic growth rate of a country or region, which is one of the essential indicators of economic development (Williams et al., 2017) [37]. The results after re-regression are shown in Table 6: Under the total sample estimation, the quality of life in the fixed-effects model will still have a positive impact on regional economic development, and the estimation results of the non-linear model are the same as the baseline regression, then it shows that the difference in the quality of life may exacerbate the gap in economic development between regions.

**Table 6:** Results of estimation with replacement of explanatory variables.

Variable	Full sample	Non-linear Model	
		Qgdp*<7*.*356*	Qgdp*≥7*.*356*
*lnlife*	0.472*** (0.015)	0.109* (0.078)	0.153*** (0.014)
*Cons*.	0.892 (1.466)	5.325*** (0.109)	5.495*** (1.328)
Control	Y	Y	Y
City FE	Y	Y	Y
Year FE	Y	Y	Y
R^2^	0.381	0.504	
Threshold P-value	-	0.00	
Obs.	245		

#### 4.4.2 Convergence effect test

Based on available studies, convergence refers to the faster a region’s economic development when it is at a lower level. Eventually, the level of economic development will converge across regions (Dellink et al., 2017) [38]. Among them, the convergence hypothesis is an integral part of the empirical study of economic growth. This test can further distinguish existing theoretical hypotheses and examine the explanatory power of theoretical models on reality (Miller and Upadhyay, 2002) [39]. In this paper, we instead use the *β-convergence* test and add the interaction term between quality of life and lagged one-period GDP per capita to the explanatory variables, from which we determine the effect of quality of life on regional economic convergence. As shown by Table 7, for model 8, the relevant control variables are not included, thus reflecting the absolute convergence of regional economic development. The coefficient of GDP per capita in the lagged period is significantly positive, which indicates that there is some convergence of economic growth among regions. In model 10, the coefficient of the lagged period is consistent with model 9. However, the coefficient of the interaction term is negative and weak, which indicates that the quality of life inhibits the convergence of regional economic development to a certain extent, in line with the above findings.

**Table 7:** Model convergence estimation results.

Variable	Model 8	Model 9	Model 10
*Qgdp*_*t-1*_	0.463*** (0.053)	1.316*** (2.986)	1.308 (2.035)
*lnlife×Qgdp*_*t-1*_	-	2.910*** (0.737)	-0.203* (0.502)
*Cons*.	9.176*** (0.092)	9.223*** (0.430)	-69.354* (33.434)
Control	N	Y	Y
R^2^	0.266	0.312	0.714
Obs	245	245	245

### 4.5 Regional heterogeneity test

**Fig 2:**
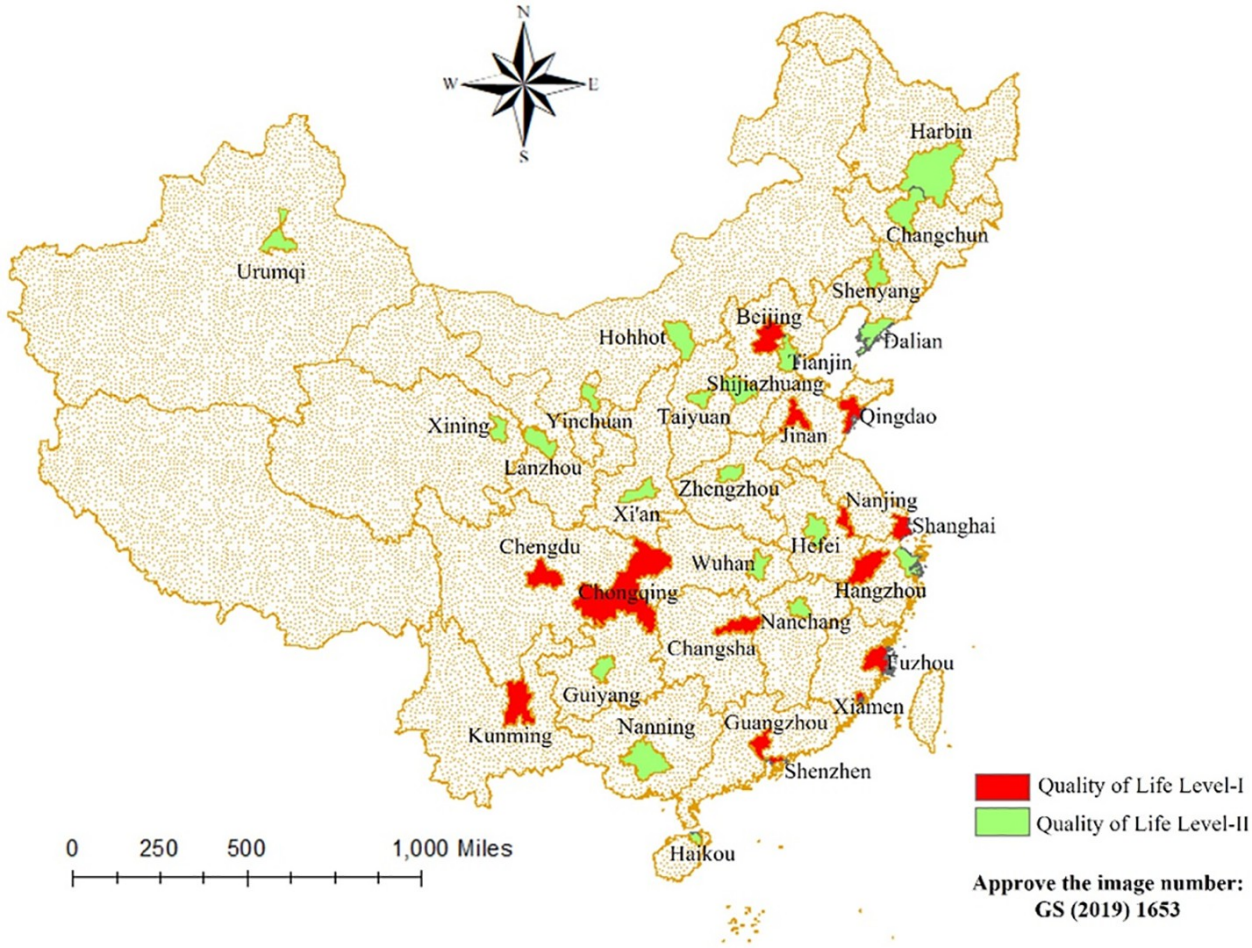
Sample selection diagram. **Note:** The base drawing is derived from the open maps of China’s national natural resources standard map service website (http://bzdt.ch.mnr.gov.cn/), the drawing number is GS (2019) 1653. This map does not change the content of the base drawing and is only used as a schematic diagram.

Regional heterogeneity analysis is a scientific method that needs to be carried out under the guidance of appropriate theories and methods and combined with corresponding statistical techniques and economic models. By comprehensively considering the impact of different factors and methods, the differences between regions can be understood better and scientific data support can be provided for policy design (Yu et al., 2019) [40]. Combined with the actual urban development in China, there are specific differences in the quality of life and economic development level among regions; thus, exploring the effect of quality of life on regional economic development under regional heterogeneity is of great practical significance. We consider the location, size, and administrative level of the cities in the sample and the average value of the Comprehensive Living Index of *the China Urban Quality of Life Report* to categorize the 35 cities in the sample into two levels. Quality of Life Level-I: Beijing, Chengdu, Fuzhou, Guangzhou, Hangzhou, Jinan, Kunming, Nanjing, Qingdao, Xiamen, Shanghai, Shenzhen, Changsha, and Chongqing; Quality of Life Level-II: Dalian, Guiyang, Harbin, Haikou, Hefei, Hohhot, Lanzhou, Nanchang, Nanning, Ningbo, Shenyang, Shijiazhuang, Taiyuan, Tianjin, Urumqi, Wuhan, Xi’an, Xining, Yinchuan, Changchun, and Zhengzhou (see Fig 2). They were included again in model 1 for regression.

According to the regression results in Table 8, from the perspective of regional heterogeneity, the overall conclusion is consistent with the overall regression results above. From models 11 to 14, the key explanatory variables *lnlife* all have a positive impact on regional economic development. It will be even easier to bring in development resources, pulling the regional economy to a certain extent. From the regional differentiation point of view, for the Quality of Life Level-I, its level of economic development is better, with advanced transportation networks, modern factories, perfect market mechanisms, etc., which can better provide a business environment and services, attract domestic and foreign investment and promote the flow of regional talent, and better adapt to the transformation of the economic structure and industrial optimization and upgrading of the needs of the economy. Therefore, the counter-effect of quality of life on regional economic development is more significant. On the contrary, in the Quality of Life Level-II, due to regional factors, social services and their development and other reasons, although the quality of life can be counteracted by economic development, the overall impact is smaller than the first level of the region, thus showing a certain degree of “weakness effect.” In summary, we conclude that when the quality of life of residents is higher, its pulling effect on regional economic growth is stronger. This will also verify hypothesis H^3^.

**Table 8:** Regional heterogeneity estimation results.

Variable	Quality of Life Level-I		Quality of Life Level-II	
	Model 11	Model 12	Model 13	Model 14
	OLS	FE	OLS	FE
*lnlife*	4.694** (2.004)	2.830** (1.229)	1.055* (1.581)	1.901* (1.084)
*lnedu*	5.847*** (0.490)	5.846*** (0.925)	2.759*** (0.528)	2.674*** (0.707)
*fore*	-0.123 (0.549)	-0.424 (0.893)	1.911* (0.807)	-0.089 (0.953)
*lnpop*	-2.356** (0.358)	-0.035* (0.478)	-2.014** (0.832)	1.377 (1.156)
*lngre*	-5.204** (1.972)	-1.141 (2.730)	-0.035 (1.459)	1.197 (1.128)
*lntsrs*	-2.923* (1.768)	0.218 (2.618)	0.754 (1.775)	-1.605* (1.315)
*lncpi*	17.103 (21.006)	5.176 (18.977)	8.768 (14.348)	8.602 (8.156)
R^2^	0.786	0.591	0.561	0.624
Obs	112	112	168	168

## 5. Conclusion and recommendations

As the world economy is under increasing downward pressure and uncertainties in global economic development increase, China’s economic development is facing a severe situation. At present, the center of gravity of China’s economic development has shifted from economic growth to improving the quality of life and wealth distribution, and “marching towards the quality of life” has become a vital development theme throughout the country. By improving the quality of life in the region, the living environment can be effectively changed to attract more talent and investment resources and provide a favorable development environment for regional economic development.

Therefore, based on the panel data model, we test the effect of quality of life on the overall development of the regional economy from an empirical point of view and further adopt the panel threshold model to estimate the extent of the role of quality of life on the economic development of different regions, according to which the quality of life will help backward regions to realize the catching-up development, or further widen the gap with the developed regions. The study results show that: First, quality of life is a new driving force for regional economic development as a whole and can effectively promote regional economic development in the context of high-quality development. Second, quality of life has a non-linear effect on regional economic development. Quality of life is more substantial in regions with higher levels of economic development and weaker in regions with lower levels of economic development. In other words, quality of life may exacerbate the disparities between regions to a certain extent. After the endogeneity treatment and robustness test, this conclusion still stands. Finally, there is significant regional heterogeneity in the inverse effect of quality of life on regional economic development. Unlike the traditional classification of east, middle and west, this paper categorizes regions based on city location, development level, quality of life level, etc. The empowering effect of higher quality of life regions on regional economic development will be more pronounced because these cities have a better financial foundation, strong policy support, and comprehensive development capability.

In summary, given the above findings, we propose the following policy recommendations: first, harmonize regional quality-of-life development factors to narrow regional quality-of-life gaps. Each region has a different geographic location, resource endowment and social development, so it is necessary to increase investment in living infrastructure according to the characteristics of each region, and to promote the development of public infrastructure, such as transportation and municipal infrastructure, while guiding the living environment to be built in an orderly manner by the market demand, to avoid duplicated construction and waste.

Second, we should focus on the balanced development of quality of life and support regions with lower quality of life in terms of policy and funding to eliminate the quality of life gap more quickly. Looking at the reality of China’s development, improving the quality of life aligns with the needs of economic and social development. We should accelerate the inclusive development of quality of life in the city, expand the living space of residents, create a high-quality urban living area, and enhance the livability of cities to provide a favorable environment for them to attract more development resources.

Third, improving the social security system to ensure the basic needs of quality of life. Social security is an essential symbol of modern life and is of great practical significance in promoting economic development and improving the quality of life. The government should take the actual social development as the starting point, all-round, multi-level, wide-area protection of the quality of life of the residents, make it standardized, institutionalized, and systematized, to lay a solid foundation for the quality of life to counteract the regional economic development.

Fourth, regions lagging in economic development should give full play to the latecomer’s advantage in terms of quality of life and concentrate on creating a high-quality living environment to reduce the cost of attracting capital and talent. They should gradually explore the path of intensive and efficient production space, livable and moderate living space, and beautiful ecological space. They should promote regional governance through planning transformation, thus creating a green and low-carbon production, and living environment.

Of course, there are still certain shortcomings in our study. (1) The construction of our system for the core variable quality of life indicators still needs to be improved. In the future, we will include specific subjects such as government and enterprises since the existing quality of life indicator system, to make the variables more representative. (2) Given the limitations of data collection and professional capacity and other factors, our research on the detailed mechanisms of quality of life and regional economic development is still weak, and in the future, we will continue to focus on this topic to improve the specificity of our research. (3) The regional scope of our study is still large, with 35 prefecture-level cities in China as representatives, and the precision still needs to be improved. So, we will further strengthen cooperation with county-level governments to enrich the research data further and enhance the study’s relevance.

## Supporting information

S1 File (PDF)
