# Supplementary material for: Quality of life and regional economic development: Evidence from China
Source: PLoS One. 2024 May 16;19(5):e0298389. doi: 10.1371/journal.pone.0298389 (PMC11098380; doi:10.1371/journal.pone.0298389)
Supplement: S1 File — (PDF) [file pone.0298389.s001.pdf]

Author's Positive Response

1. Dr. Yuhu Cui

Re:ACTION REQUIRED for [PONE-D-23-07277] 安全浏览模式 精简信息

发件人：崔<cui19982020@163.com>
收件人：plosone<plosone@plos.org>
抄送人：zeedyork<zeedyork@163.com>
时　间：2024年03月26日 19:03 (星期二)
附　件：3个 (0315-Author statement.docx 等) 查看附件

发送状态：发送成功 查看详情

翻译成中文

【阿里云】2核4G仅需68.66元/月 立即抢购

Dear Editor:

Greetings! I have reviewed the latest revised manuscript and agree with all the changes made by the corresponding author.

However, for the list of authors, probably because of the time lag before and after the revision, the latest results are still not reflected in the content of the email, and I would like to give you feedback on the specific situation as follows:

Because some members of the team (Dr. Chunlei Shen and Prof. Lin Yang) have recently changed their new work organizations. Therefore, after Dr. Shen and Prof. Yang took the initiative to apply, the team removed them from the author list with full consideration of the name ownership confidentiality agreement signed between them and their new units.

After the decision of all authors, the final order of authors： Yuhu Cui, Hu Tian, Dong An, Yonghua Jia*.

Please refer to the attachment for the latest revised manuscript information, thank you!

We are very pleased that the article can be published in Plos One, and now various things are ready, looking forward to your reply!

Best wishes.

Dr. Cui

2. Dr. Hu Tian

Re:ACTION REQUIRED for [PONE-D-23-07277]

发件人：田虎<tianhuouc@163.com>
收件人：plosone<plosone@plos.org>　收起
抄送人：zeedyork<zeedyork@163.com>
时　间：2024年03月26日 17:39 (星期二)
附　件：0315-Author stateme... 0315-plos-one-chang... 0315-PONE-D-23-07...
共3个附件

发送状态：**发送成功** 查看详情

Dear Editor:

Greetings! I have reviewed the latest revised manuscript and agree with all the changes made by the corresponding author.

However, for the list of authors, probably because of the time lag before and after the revision, the latest results are still not reflected in the content of the email, and I would like to give you feedback on the specific situation as follows:

Because some members of the team (Dr. Chunlei Shen and Prof. Lin Yang) have recently changed their new work organizations. Therefore, after Dr. Shen and Prof. Yang took the initiative to apply, the team removed them from the author list with full consideration of the name ownership confidentiality agreement signed between them and their new units.

After the decision of all authors, the final order of authors： Yuhu Cui, Hu Tian, Dong An, Yonghua Jia*.

Please refer to the attachment for the latest revised manuscript information, thank you!
We are very pleased that the article can be published in Plos One, and now various things are ready, looking forward to your reply!

Best wishes.
Dr. Tian

3. Dr. Dong An

Re:ACTION REQUIRED for [PONE-D-23-07277] 安全浏览模式

发件人: 盛文倩<swq17861506797@163.com>
收件人: plosone<plosone@plos.org>
抄送人: zeedyork<zeedyork@163.com>
时 间: 2024年03月26日 19:18 (星期二)
附 件: 3个 (0315-Author statement.docx 等) 查看附件

发送状态: 邮件发送中 查看详情
翻译成中文

【阿里云】2核4G仅需68.66元/月 立即抢购

Dear Editor:

Greetings! I have reviewed the latest revised manuscript and agree with all the changes made by the corresponding author.

However, for the list of authors, probably because of the time lag before and after the revision, the latest results are still not reflected in the content of the email, and I would like to give you feedback on the specific situation as follows:

Because some members of the team (Dr. Chunlei Shen and Prof. Lin Yang) have recently changed their new work organizations. Therefore, after Dr. Shen and Prof. Yang took the initiative to apply, the team removed them from the author list with full consideration of the name ownership confidentiality agreement signed between them and their new units.

After the decision of all authors, the final order of authors: Yuhu Cui, Hu Tian, Dong An, Yonghua Jia*.

Please refer to the attachment for the latest revised manuscript information, thank you!

We are very pleased that the article can be published in Plos One, and now various things are ready, looking forward to your reply!

Best wishes.

Dr. An

4. Dr. Chunlei Shen

Re:ACTION REQUIRED for [PONE-D-23-07277]

发件人: [illegible] <ma5427889@vip.qq.com>
时 间: 2024年3月26日 (星期二) 下午7 : 56
收件人: plosone <plosone@plos.org>
抄 送: zeedyork <zeedyork@163.com>
附 件: 3 个 (0315-Author statement.docx...)

纯文本 |

发送状态: 正在投递 [关闭] [刷新] 什么是发送状态?

收件人	投递状态	时间
plosone@plos.org		2024年3月26日(星期二) 晚上7:56
zeedyork@163.com		2024年3月26日(星期二) 晚上7:56

邮件可翻译为中文 立即翻译

Dear Editor:
Greetings! I have reviewed the latest revised manuscript and agree with all the changes made by the corresponding author.
However, for the list of authors, probably because of the time lag before and after the revision, the latest results are still not reflected in the content of the email, and I would like to give you feedback on the specific situation as follows:
Because some members of the team (Dr. Chunlei Shen and Prof. Lin Yang) have recently changed their new work organizations. Therefore, after Dr. Shen and Prof. Yang took the initiative to apply, the team removed them from the author list with full consideration of the name ownership confidentiality agreement signed between them and their new units.
After the decision of all authors, the final order of authors: Yuhu Cui, Hu Tian, Dong An, Yonghua Jia*.
Please refer to the attachment for the latest revised manuscript information, thank you!
We are very pleased that the article can be published in Plos One, and now various things are ready, looking forward to your reply!
Best wishes.
Dr. Shen

5. Prof. Lin Yang

Re:ACTION REQUIRED for [PONE-D-23-07277] 安全浏览模式

发件人: Guo<ghw0726@163.com>
收件人: plosone<plosone@plos.org>
抄送人: zeedyork<zeedyork@163.com>
时 间: 2024年03月26日 19:07 (星期二)
附 件: 邮件大小超过1M，普通附件未被保存，已成功发出的邮件不受影响 (马上保存 | 设置自动保存)

发送状态: 发送成功 查看详情
翻译成中文

【阿里云】2核4G仅需68.66元/月 立即抢购

Dear Editor:

Greetings! I have reviewed the latest revised manuscript and agree with all the changes made by the corresponding author.

However, for the list of authors, probably because of the time lag before and after the revision, the latest results are still not reflected in the content of the email, and I would like to give you feedback on the specific situation as follows:

Because some members of the team (Dr. Chunlei Shen and Prof. Lin Yang) have recently changed their new work organizations. Therefore, after Dr. Shen and Prof. Yang took the initiative to apply, the team removed them from the author list with full consideration of the name ownership confidentiality agreement signed between them and their new units.

After the decision of all authors, the final order of authors: Yuhu Cui, Hu Tian, Dong An, Yonghua Jia*.

Please refer to the attachment for the latest revised manuscript information, thank you!

We are very pleased that the article can be published in Plos One, and now various things are ready, looking forward to your reply!

Best wishes.

Prof. Yang
